# Treatment of tibial bone defects: pilot analysis of direct medical costs between distraction osteogenesis with an Ilizarov frame and the Masquelet technique

**DOI:** 10.1007/s00068-022-02162-z

**Published:** 2022-11-28

**Authors:** Nikolaos K. Kanakaris, Paul J. Harwood, Ruben Mujica-Mota, Ganesh Mohrir, George Chloros, Peter V. Giannoudis

**Affiliations:** 1 Leeds Major Trauma Centre, Leeds General Infirmary, Leeds Teaching Hospitals NHS Trust, Clarendon Wing, Level D, Leeds, LS13EX West Yorkshire UK; 2 Major Trauma Centre, Leeds Teaching Hospitals NHS Trust, Leeds, UK; 3 Academic Unit of Health Economics, University of Leeds, Leeds, UK; 4 Department of Trauma and Orthopaedics, Leeds Teaching Hospitals NHS Trust, Leeds, UK; 5 Academic Department of Trauma and Orthopaedics, University of Leeds, Leeds, UK

**Keywords:** Bone defect, Cost analysis, Tibia, Masquelet technique, Distraction osteogenesis, Ilizarov circular frame

## Abstract

**Purpose:**

The cost implications of limb reconstruction techniques have not been adequately investigated. Aim of this pilot study was to compare the direct medical cost of tibial bone defects managed with distraction osteogenesis–Ilizarov method (ILF), or with Masquelet technique (MIF).

**Methods:**

Data of 20 random patients treated in a single centre were analysed. Inclusion criteria included acute tibial defects, or post-debridement of nonunions with complete follow-up and successful union. The endpoint of clinical efficacy was the time-to-defect union. Comparisons were made between equally sized subgroups (ILF vs. MIF).

**Results:**

The average defect length was 5.6 cm (2.6–9.6 cm). The overall cost of 20 cases reached £452,974 (mean £22,339, range £13,459–£36,274). Statistically significant differences favoring the MIF were found regarding the average time-to-union; number of surgeries, of admissions and follow-up visits, as well as the mean intraoperative cost (£8857 vs. £14,087). These differences lead to significant differences of the mean cost of the overall treatment (MIF £18,131 vs. ILF £26,126). Power analysis based on these data indicated that 35 patients on each group would allow detection of a 25% difference, with an alpha value of 0.05 and probability (power) of 0.9.

**Conclusions:**

The results and analysis presented highlight factors affecting the high financial burden, even in a best-case scenario, this type of surgery entails. Larger pivotal studies should follow to improve the cost efficiency of clinical practice.

## Introduction

Successful management of bone defects remains a significant clinical challenge. Whether attributed to acute bone loss (occurring in 11.4% of severe open fractures) [1], resections for bone tumors, nonunions, or infections [2], they require considerable surgical expertise, patient compliance, multidisciplinary pathways, and consume significant resources [3, 4]. The most common site of bone loss is the tibia with a number of clinical series reporting different management strategies and outcomes [2, 5, 6].

Contemporary treatments include distraction osteogenesis (using circular fine wire fixators, monolateral external devices, or lengthening nails), vascularized bone grafts, the Masquelet technique, the use of titanium cages or even amputation under certain circumstances [2, 3, 5–7]. The relative rarity of this clinical problem and the complexity of its management have led to centralization of this work to specialized limb reconstruction groups. Having most of these techniques readily available, it offers flexibility, efficiency, individualized care, and theoretically limits the associated socioeconomic burden [5, 7–9].

The cost implications of these different techniques have not been adequately explored [10–12]. Under the current strenuous health economic climate [13], and the increasing complexity of medical care, sustainable provision of limb reconstruction services dictates appropriate reimbursement strategies based on reliable cost analyses [10, 14, 15].

The primary aim of this study was to produce a pilot cost analysis on tibial bone defects, to show the feasibility of collecting the data for conducting robust and detailed cost analysis and inform future evaluations of costs and effectiveness. Secondary endpoints were a) to compare the direct cost between bone transport using a fine wire circular fixator (ILF) and the Masquelet technique using internal fixation (MIF) and b) to compare the direct cost between acute tibial bone loss of open fractures vs. cases with secondary bone loss generated during the treatment of tibial nonunions.

## Patients and methods

Prospectively collected data from a single centre acting as a level 1 trauma centre and regional referral unit for limb reconstruction were analysed. Exclusion criteria included patients below 18 years of age, tibial defects of different causation (tumor or otherwise), patients treated with other techniques, or who did not heal their defect or lost to follow up. The method that each of the patients was treated was decided at the time based on the consensus reached during departmental clinical governance meetings, and individual patient’s preference during the informed consent process. Patients with adverse outcomes were excluded, because we wished to assess the cost of both techniques in a best-case scenario. We felt that managing treatment failure in these patients will significantly increase cost and this would need investigation in a larger patient group to be meaningful.

To reduce selection bias, the first five patients in alphabetic order of their surname, that received treatment at the acute (ILFa–MIFa) or nonunion (ILFn–MIFn) settings with either technique were included for further assessment of their direct costs till completion of follow-up.

Data collected included demographics, comorbidities (Charlson’s score [16]), surgical risk (ASA score [17]), severity of trauma (ISS [18]), fracture type (AO/OTA [19]) and Gustilo–Anderson systems [20, 21] for the open fractures. The size of all defects was recorded at the operative notes of the final debridement, and further classified using the Solomin–Slongo system [22]. The duration of surgery, the implants used, administered blood products, laboratory tests performed, imaging investigations, length-of-hospital stay (LOS), visits to the outpatient clinics, time-to-union and time-to-discharge from further follow-up were collected in an excel database. Time-to-union was defined as the time till the first mention of a healed defect by the treating surgeon to the patient’s records and verified by the radiology reports.

To define the direct medical costs, we utilised the financial records of several clinical service units. These included the records of trauma-related specialties, operating theatres, blood bank, outpatient clinic and patient transport departments. Data from the 2019/20 National Tariff [23], the BNF (British National Formulary) [24], as well as from the price list of all devices and implants from industry partners were collected. All costs were adjusted for inflation to the United Kingdom’s 2020 consumer price index at a rate of 2.2% [25]. The detailed template of the exact prices per item are presented in Tables 1 and 2.

**Table 1:** Direct medical costs (inhospital and outpatient stay, OR procedures, medications)

Phase	Items	Cost	Source	Phase	Items	Cost	Source
Inhospital stay	Standard ward hospital stay per day	£241.00	TRS CSU	Intraoperative costs	OR trauma (per minute)	£319.00	NHS England. 2019/20 National Tariff Payment System
	High observations ward hospital stay per day	£412.00	ACC CSU		Consultant time in OR (per minute)	£49.00	
Laboratory tests	Full blood count (FBC)	£2.65	NHS England. 2019/20 National Tariff Payment System		Registrar time in OR (per minute)	£30.00	
	Biochemical tests (U&Es)	£2.12			Sterilisation cost per kit	£75.00	OR THEATRES CSU
	Clotting tests	£3.83		Outpatient clinics costs	Fup f2f visit	£104.00	OPC CSU
	group and save	£8.00			First visit	£128.00	
	cRBC transfusion	£781.00	BLOOD BANK		Transport W1–2	£85.00	
Imaging tests	plain X-ray	£25.00	NHS England. 2019/20 National Tariff Payment System		Transport T1–2	£77.00	
	CT scan	£118.00			Transport SC	£43.00	
	MRI scan	£157.00		Antibiotic therapies	Vancomycin iv 1gr vial	£12.50	BNF 2020 NICE ORG UK^27^
Intraoperative costs	OR Trauma (per minute)	£319.00	NHS England. 2019/20 National Tariff Payment System		Gentamycin iv 40 mg/1 ml ampoule	£1.20	
	Consultant time in OR (per minute)	£49.00			Flucloxacillin iv 500 mg vial	£1.25	
	Registrar time in OR (per minute)	£30.00			Flucloxacillin p.os 500 mg capsule	£0.25	
	Sterilisation cost per kit	£75.00	Operating Theatres CSU		Co-amoxiclav iv 1.2 g vial	£1.60	
	Palacos^®^ MV G cement × 1 mix	£44.03			Teicoplanin iv 400 mg vial	£12.00	
	Preoperative antibiotic prophylaxis at induction (open fractures)	£26.40			Ciprofloxacin p.os 750 mg tablet	£0.80	
	Preoperative antibiotic prophylaxis at induction (other)	£8.40	BNF 2020 NICE ORG UK^27^		Ciprofloxacin iv 400 mg/200 ml vial	£19.92	
					Piperacillin/tazobactam 2gr/250 mg vial	£9.95	
VTE prophylaxis	Tinzaparin 4500units prefilled disposable sc injection	£3.56	BNF 2020 NICE ORG UK^27^		Linezolid p.os 500 mg tablet	£31.24	
Painkillers	Codeine phosphate 30 mg tablet	£1.23	BNF 2020 NICE ORG UK^27^		Rifampicin p.os 150 mg capsule	£54.65	
	Paracetamol 500 mg tablet	£0.20			Clindamycin p.os 75 mg capsule	£0.31	

ACC = acute critical care; BNF = British National Formulary; cRBC = concentrated red blood cell transfusion; CSU = Clinical Service Unit; g = gram; G = gentamicin; iv = intravenous administration route; LTHT = Leeds Teaching Hospitals Trust; mg = milligram; MRI = magnetic resonance imaging; MV = medium viscosity; NICE = national institute of health and care excellence; OR = operation room; p.os = oral administration route; SC = saloon care transport vehicle; T1/2 = tail lift transport vehicle; TRS = trauma-related services; UK = United Kingdom; VTE = venous thromboembolic events; W1/2 = wheelchair transport vehicle

**Table 2:** Direct medical cost of implants and bone graft substitutes

Description	Cost		Source		Description		Cost		Source		Description		Cost		Source		Description		Cost		Source	
ALPS^®^ distal tibial anterolateral plate	£1065.00		Zimmer biomet UK		Half ring 160 mm		£276.17		Smith and nephew UK		Expert tibial nail PROTECT		£2024.00		DePuy Synthes UK		Stimulan bone graft 10 ml		£490.00		Biocomposites UK	
ALPS^®^ distal tibial medial plate	£942.00				Thick Italian nut		£24.91				Guide wire 3.2 mm		£29.92				Cerament V/G 10 ml		£1500.00		BoneSupport UK	
ALPS^®^ distal fibula anatomical plate	£628.00				Thread rod 150 mm		£74.04				Ball tip guide wire		£187.00				Cerament V/G 5 ml		£1000.00			
Locking cortical screw	£74.00				Wire with stopper 400 mm × 12		£1028.96				Locking screw L144		£43.71				Bonalive 10 ml		£467.40		Bonalive biomaterials	
Non-locking cortical screw	£24.00				Wire fixed bolt slotted		£101.33				Cancellous locking screw L50		£63.14				Bonalive 5 ml		£820.00			
Kwire 1.6 × 150 mm	£48.00				Foot composite ring		£339.69				End cap		£41.60				BMAC biocue 60 ml		£466.00		Zimmer biomet UK	
Drill bit 2.5 mm	£88.00				Kits		× 4				Drill bit 3.2 mm calibr L340		£156.17				PRP recover 60 ml		£278.00			
Drill bit 2.7 mm	£114.00				Average 5 ring construct		£4892.70				Drill bit 4.2 mm calibr L135		£120.97				RIA system harvest average use		£1285.87		Procurement department	
Kits	× 3				Average 6 ring construct		£5519.08				Outer protection sleeve (suprapatellar)		£30.88									
evos 2.7/3.5 mm P/A M-D plate	£514.00		smith & nephew UK		average 7 ring construct		£6096.33		stryker UK		kits		× 4		zimmer biomet UK		VACPAC average use		£146.40		3 M KCI—TRS CSU LTHT	
evos 2.7/3.5 mm L-D fibula PL plate	£474.30				Hoffman 2 clamp rod to rod		£549.00				Versanail tibia		£515.00									
3.5 mm locking screw	£95.10				Hoffman 2 clamp pin to rod		£527.00				3.2 × 444 mm threaded guide pin		£54.00									
4.7 mm osteopenic screw	£19.70				8 mm rod		£74.00				ball nose guide wire 100 cm		£146.00									
3.5 mm non-Locking cortical screw	£24.90				Hoffman 3 clamp rod to rod		£632.00				4.4 mm x 286 mm drill bit		£226.00									
2.7 mm locking screw	£50.90				Hoffman 3 clamp pin to rod		£606.00				3.8 mm x 150 mm drill bit		£230.00									
2.7 mm non locking screw	£29.90				11 mm rod		£85.00				end cap		£39.00									
drill bit 2.5 mm	£86.50				5 mm × 180 apex pin		£88.00				5.5 mm solid cortical screw		£41.00									
drill bit 2.0 mm	£129.20				3.2 mm drill bit		£51.00				4.5 mm solid cortical screw		£26.00									
kits	× 3				kits		× 3				kits		× 4									

*ALPS*^®^ advanced locking plating system, *BMAC* bone marrow aspirate concentrate, *mm* millimetre, *PRP* platelet rich plasma

Descriptive statistical methods (two sample *t* test) have been utilised. Independent samples t tests were performed to compare the means in all parameters, following log transformation of the recorded values. We studied the complete follow-up period and also different timepoints for (a) ILF vs. MIF groups; (b) acute vs. nonunion defects. The alpha value of 0.05 was used as the cutoff for statistical significance. We considered adjusting for baseline clinical differences in our analysis of cost differences, but the small numbers of patients in each subgroup at this pilot study prevented us from doing so.

## Results

Data from 20 patients were analysed: five patients of each of the groups (ILFa, ILFn, MIFa, and MIFn). The overall direct medical cost treating these 20 tibial bone defects [mean length of 5.6 cm (range 2.6–9.5)] was £452,974. Patient and defect characteristics are shown in Table 3.

**Table 3:** Characteristics of the overall group of patients with tibial defects, with comparison between different treatment methods (distraction osteogenesis with an Ilizarov frame) or Masquelet internal fixation technique (induced membrane); as well as comparison between acute defects vs. nonunion or debridement due to fracture-related infection

Parameters	Overall	Distraction osteogenesis protocol (ILF)	Induced membrane protocol (MIF)	Difference between means (ILF minus MIF)	*p* value	Acute defect	Nonunion defect	Difference between means (acute minus nonunion defects)	*p* value
Number of patients	**20**	**10**	**10**	*n/a*	*n/a*	**10**	**10**	*n/a*	*n/a*
Gender ratio	**14/6**	**9/1**	**5/5**	*n/a*	**< *0.001***	**7/3**	**7/3**	*n/a*	*1*
(M/F)									
Mean age	**39 years**	**38 years**	**40 years**	*- 2 years*	*0.738*	**39 years**	**38 years**	*1 year*	*0.867*
(SD, median, range)	(13.17, 34, 20–64)	(12.22, 38, 23 to 56)	(14.05, 34, 20 to 64)			(14.14, 38, 20 to 57)	(12, 33, 26 to 64)		
Mean ISS	**12**	**12**	**13**	*-1*	*0.722*	**12**	**n/a**	*n/a*	*n/a*
(SD, median, range)	(6.23, 9, 9–27)	(5.2, 9, to 9 to 22)	(7.04, 9, 9 to 27)			(6.23, 9, 9 to 27)			
Mean Charlson's score	**0.53**	**0.1**	**1**	*-0.9*	***0.025***	**0.5**	**0.56**	*-0.06*	*0.880*
(SD, median, range)	(0.88, 0, 0–3)	(0.3, 0, 0 to 1)	(1.05, 1, 0 to 3)			(0.92, 0, 0 to 3)	(0.83, 0, 0 to 3)		
Mean ASA score	**1.37**	**1.2**	**1.56**	*-0.36*	*0.170*	**1.5**	**1.22**	*0.28*	*0.280*
(SD, median, range)	(0.58, 1, 1–3)	(0.4, 1, 1 to 2)	(0.68, 1, 1 to 3)			(0.67, 1, 1 to 3)	(0.42, 1, 1 to 3)		
Mean length of defect	**5.59 cm**	**5.96 cm**	**5.18 cm**	*0.78 cm*	*0.189*	**5.08 cm**	**6.15 cm**	*- 1.07 cm*	*0.433*
(SD, median, range)	(1.9, 5.15, 2.6–9.52)	(1.7, 5.21, 3.3 to 8.3)	(2.02, 4.26, 2.6 to 9.52)			(1.31, 5.03, 2.6 to 7.36)	(2.25, 5.7, 3.3 to 9.52)		
Mean follow up period	**30.52 months**	**33.18 months**	**27.57 months**	*5.61 months*	*0.123*	**23.57 months**	**38.25 months**	*- 14.68 months*	***0.04***
(SD, median, range)	(17, 26.03, 12–74)	(17.01, 26.73, 14.97 to 74)	(16.5, 21.83, 12 to 62.43)			(17.59, 18.4, 12.6 to 74)	(12.38, 34.8, 12 to 62.43)		
Mean time to union	**12.91 months**	**15.5 months**	**10.03 months**	*5.47 months*	***0.003***	**11.2 months**	**14.8 months**	*- 3.6 months*	***0.048***
(SD, median, range)	(4.38, 12.4, 4.6–22.2)	(3.83, 15.55, 8.3 to 22.2)	(2.92, 9.73, 4.6 to 15.9)			(4.07, 9.22, 6.97 to 17.27)	(3.5, 13.07, 4.6 to 22.2)		
Mean Healing Index	2.23	2.35	1.99	*-0.36*	*0.276*	2.11	2.37	*− 0.26*	*0.418*
(SD, median, range)	(0.71, 2.03, 1.1–3.76)	(0.76, 2.11, 1.5 to 3.76)	(0.67, 1.71, 1.1 to 2.85)			(0.67, 1.89, 1.35 to 3.52)	(0.73, 2.42, 1.1 to 3.76)		
Mean no of surgeries	**3**	**4**	**3**	*1*	***0.049***	**3**	**4**	*-1*	*0.102*
(SD, median, range)	(1.27, 3, 2–7)	(1.3, 4, 2 to 7)	(0.67, 3, 2 to 4)			(0.9, 3, 2 to 5)	(1.57, 3, 2 to 7)		
Mean no of admissions	**3**	**3**	**2**	*1*	***0.026***	**2**	**3**	*-1*	***0.036***
(SD, median, range)	(1.03, 2, 1–5)	(1.17, 4, 1 to 5)	(0.32, 2, 2 to 3)			(0.92, 2, 1 to 4)	(1.05, 3, 2 to 5)		
Mean LOS till union	**20 days**	**21 days**	**19 days**	*2 days*	*0.872*	**18 days**	**22 days**	*-4 days*	*0.342*
(SD, median, range)	(7.69, 18, 8–36)	(8.92, 20, 8 to 36)	(5.48, 18, 10 to 28)			(4.96, 17, 10 to 28)	(9.19, 20, 8 to 36)		
Mean cost of inpatient stay	**£7024**	**£6693**	**£7392**	− *£699*	*0.467*	**£7121**	**£6916**	*£205*	*0.749*
(SD, median, range)	(4017.88, 6248, 2010.74–19,349.86)	(3408.84, 6041, 2010.74 to 13,289.08)	(4573, 6248, 3499.45 to 19,349.86)			(4854.87, 5041, 3499.45 to 19,349.86)	(2805.31, 7851, 2010.74 to 12,640.33)		
Mean cost of procedures	**£11,610**	**£14,087**	**£8857**	*£5230*	***0.004***	**£11,199**	**£12,066**	− *£867*	*0.597*
(SD, median, range)	(3891.68, 10,177, 6222.84–20,333.93)	(3595.78, 14,688, 8318.9 to 20,333.93)	(1790.75, 8736, 6222.84 to 15,991.72)			(2992.26, 10,892, 6222.84 to 15,598.08)	(4650.64, 9429, 7213.85 to 20,333.93)		
Mean cost of outpatient Fup	**£3775**	**£5240**	**£2147**	*£3093*	**< *0.001***	**£3066**	**£4564**	− *£1498*	*0.172*
(SD, median, range)	(2212.9, 3324, 821.2–8771.85)	(1954.71, 5094, 1947 to 8771.85)	(1027.97, 2034, 821.2 to 3930.45)			(2342.96, 2010, 821.2 to 8771.85)	(1748.49, 3930, 898 to 8035.16)		
mean Overall Cost	**£22,339**	**£26,126**	**£18,131**	*£7995*	***0.025***	**£21,385**	**£23,399**	− *£2014*	*0.475*
(SD, median, range)	(7142.82, 20,469, 13,459.45–36,273.98)	(7128.58, 27,096, 14,763.77 to 36,273.98)	(4195.51, 16,788, 13,459.45 to 29,530.05)			(5796.33, 21,504, 13,459.45 to 29,618.24)	(8260.97, 18,208, 14,763.77 to 36,273.98)		
Mean cost per cm of defect	**£4295**	**£4612**	**£3944**	*£668*	*0.534*	**£4174**	**£4208**	− *£34*	*0.865*
(SD, median, range)	(1423.47, 4300, 1736.96–7447.75)	(1423.58, 4362, 1974.88 to 7447.75)	(1338.15, 3972, 1736.96 to 7030.96)			(1114.61, 4288, 1959.75 to 6044.54)	(1687.96, 4424, 1736.96 to 7447.75)		

*ASA* American Society of Anaesthesiologists physical status score, *Fup* follow up, *Healing index* time to union in months/length of defect in cm, *LOS* length of stay, *ISS* injury severity score, *No* number, *SD* standard deviation

According to the Solomin–Slongo system, four defects were type C1, three B2, and one B3, D2 and D3 [22]. According to the AO/OTA system [19], there were eight 43.A3, four 42.B3, three 42.B2, two 42.A2, and one fracture for each of the 41.A3, 42.A3, and 42.C2 types. The ten patients with an acute defect (ILFa and MIFa) all had a type-III [21] open fractures. The ten nonunion defects (ILFn and MIFn) were proven infected in seven. Systemic antibiotic treatment ranged between 6 and 9 weeks. The overall cost of the antibiotic therapy reached £6058 with a mean of £865 (range £54–£1969).

Wound vacuum-assisted closure was utilised in five open type-III-B fractures. Microsurgical soft-tissue reconstruction was required in 7 with an acute (4 × ILFa and 3 × MIFa) and in 4 with a nonunion defect (2 × ILFn and 2 × MIFn). Definitive orthoplastic surgery at the same seating was performed in 50% of the cases (1 × ILFa, 2 × MIFa, × 1 ILFn, and 1 × MIFn). The other 50% had first a free flap, and on a different day their definitive fixation (3 × ILFa, × 1 ILFn and × 1 MIFn) after a mean of 11.4 days (range 9–15).

The Masquelet staged protocol that we followed has been previously described. [5, 26] The mean period between the two stages was 61 days (range 42–128). The polymethacrylate cement spacer was combined with antibiotics (vancomycin 2 g and 40 mg of gentamycin per mix). Internal fixation was used at the first stage in five cases in the form of two reamed nails and three plate fixations. In the other five MIF cases, an external fixator was placed at the first stage, which at the second stage was revised to plate fixation. The reamer irrigator aspiration system (RIA^®^ of DePuy Synthes)[27] was utilised in nine patients to harvest bone graft at the second stage. In four MIF cases, composite grafts were utilised combining the RIA harvest with bone-marrow-aspirate-concentrate and platelet-rich-plasma. In one MIFa case, iliac-crest-autologous-graft (ICAG) was combined with BMP2. The mean time-to-union index (ratio of time from first debridement till the verification of defect union in months, divided by the length-of-defect in cm) was 1.8 (range 1.1–2.9).

In one of the MIFn patients, chronic donor-site pain developed at the trochanteric area. Another patient 2 years after completion of union of his defect developed a relapse of the infection, which was managed effectively with plate removal and pathogen specific antibiotic therapy.

The bone transport patients were operated according to the principles of the Ilizarov technique [28, 29]. The mean period between frame application and corticotomy (single-level percutaneous) was 24.8 days (range 0–94). Transport was initiated after a latent period of 10–12 days with a distraction rate of 0.5–1 mm/day. Prior to removal, the frame was dynamized to verify the mechanical stability of the regenerate. The mean healing index (ratio of time from frame application till the date of its removal in months, divided by the length-of-defect in cm) was 2.1 (range 1.5–3.8).

One patient required a second corticotomy due to premature consolidation, a further patient required minor frame revision due to broken wires. Three ILF patients experienced a docking site refracture following frame removal (at 3–5 month postremoval). Two were successfully managed with a Sarmiento cast, and one with additional surgery (nailing). Three patients had recurrent pin-track infections that settled with oral antibiotics. An additional patient developed a delayed pin-track infection post union which required debridement, local antibiotics and a local fasciocutaneous flap. Only one frame patient had his frame removed at the outpatient clinics under gas-and-air anaesthesia. The rest required a day-case admission. Persistent neuropathic pain and acceptance of a 2 cm shortening in one patient, ankle stiffness in two at final follow-up were also recorded as associated complications.

Between the ILF and the MIF groups, no statistically significant difference was noted in regard to their mean age, ISS, ASA-score, length-of-defect, associated soft-tissue reconstruction procedures, the overall LOS and follow-up, and the cost of in-hospital stay. Statistically significant difference favoring the ILF group was found to the comorbidity index (*p* = 0.02), as well as to the gender ratio (*p* < 0.001). Results favoring the MIF group were found in regard to the mean time-to-union (*p* = 0.03), the number of procedures (*p* = 0.049), of admissions (*p* = 0.026), the operative room (OR) cost (*p* = 0.004), the cost of outpatient follow-up (*p* < 0.001), and the cost of the overall treatment (*p* = 0.025), Table 3.

The total cost in the MIF was £192,711, compared to £260,263 of the ILF, or else 26% lower for the same number of random patients with successful eventual defect union. For the ILF patients, 54% of the overall cost was related to the OR, the 25% to the inpatient stay, and 21% to the outpatient follow-up. Respectively, for the MIF patients, the costs at these different stages were 49%, 41%, and 10%). There was statistically significant difference favoring the MIF group on the average cost at the OR (*p* = 0.004) and the outpatient(*p* < 0.001) phases, Table 3.

When comparing the acute vs. the nonunion defect groups, there were no statistically significant differences for the majority of parameters. Exceptions to this were the mean number of admissions(*p* = 0.036), the time-to-union(*p* = 0.048), and the follow-up period(*p* = 0.04), which were all higher for the nonunion defects, Table 3.

Further subgroup analysis (Table 4) identified no statistically significant difference between the direct medical costs when the Ilizarov technique was used for an acute or a nonunion tibial defect. When the Masquelet groups were compared, the mean time-to-union (8 vs. 13 months), as well as the overall follow-up period (15 vs. 44 months), and the cost of outpatient follow-up (£1368 vs. £3122) were significantly higher when the defect was associated with a nonunion.

**Table 4:** Head-to-head comparison between the four subgroups (ILFa, ILFn, MIFa, and MIFn)

Subgroups	Mean age	Mean Charlson's index	Mean ASA	CAUSE	Mean ISS	Mean defect size	Mean time to union	Mean healing Index	Mean fup	Mean LOS till union	Mean no of admissions	Mean no of procedures	Mean cost overall	Mean cost inpatient stay	Mean cost OR	Mean cost outpatient clinic fup	Mean cost per cm of defect
	(SD, median, range)	(SD, median, range)	(SD, median, range)		(SD, median, range)	(SD, median, range)	(SD, median, range)	(SD, median, range)	(SD, median, range)	(SD, median, range)	(SD, median, range)	(SD, median, range)	(SD, median, range)	(SD, median, range)	(SD, median, range)	(SD, median, range)	(SD, median, range)
ILFa	40 years	0.2	1.4	Motorcycle (× 3) Pedestrian (× 1) Car occupant (× 1)	12	5.42 cm	14.49 m	2.41	32.45 m	19 days	3	4	£25,364.00	£6906.00	£13,695.00	£4763.00	£4776.16
	*(14.57, 42, 23 to 56)*	*(0.4, 0, 0 to 1)*	*(0.49, 1, 1 to 2)*		*(5.2, 9, 9 to 22)*	*(0.81, 5.15, 4.76 to 7)*	*(3.19, 15.77, 8.3 to 17.27)*	*(0.7, 2.03, 1.58 to 3.52)*	*(21.17, 23.67, 14.97 to 74)*	*(3.31, 20, 13 to 20)*	*(1.17, 3, 1 to 4)*	*(0.63, 4, 3 to 5)*	*(3695.96, 24,621, 20,468.6 to 29,618.24)*	*(3270.29, 5806, 4199.43 to 13,289.08)*	*(1640.32, 14,335, 11,606.84 to 15,598.08)*	*(2220.27, 4377, 1947 to 8771.85)*	*(959.43, 4300.13, 3517.22 to 6044.54)*
MIFa	38 years	0.8	1.6	Fall > 2 m (× 3) Pedestrian (× 1) Car occupant (× 1)	13	4.74 cm	7.91 m	1.99	14.69 m	17 days	2	3	£17,407.00	£7336.00	£8703.00	£1368.00	£3972.36
	*(13.57, 34, 20 to 57)*	*(1.17, 0, 0 to 3)*	*(0.8, 1, 1 to 3)*		*(7.04, 9, 9 to 27)*	*(1.6, 4.26, 2.2 to 7.6)*	*(1.17, 7.1, 6.97 to 9.97)*	*(0.48, 1.94, 1.35 to 2.73)*	*(3.58, 13, 13 to 21.83)*	*(6.01, 16, 10 to 28)*	*(0, 2, 2 to 2)*	*(0.49, 3, 2 to 3)*	*(4677.6, 15,767, 13,459.45 to 26,461.9)*	*(6029.25, 4480, 3499.45 to 19,349.86)*	*(1660.28, 9813, 6222.84 to 10,177.25)*	*(532.89, 1109, 821.2 to 2034.42)*	*(1114.07, 3972.46, 1959.75 to 5176.71)*
*p* value	*0.754*	*0.153*	*0.511*	*N/A*	*0.722*	*0.332*	*0.005*	*0.137*	*0.026*	*0.382*	*0.024*	*0.001*	*0.017*	*0.813*	*0.013*	*0.002*	*0.274*
ILFn	35 years	0	1	FRI (× 3) NU (× 2)	N/A	6.5 cm	16.5 m	2.3	33.92 m	23 days	4	4	£26,889.00	£6481.00	£14,480.00	£5717.00	£4448.14
	*(8.47, 33, 26 to 47)*	*0*	*(0, 1, 1 to 1)*			*(2.12, 8.1, 3.3 to 8.3)*	*(4.14, 15.33, 12.1 to 22.2)*	*(0.8. 2.2, 1.5 to 3.76)*	*(11.39, 27.6, 25.47 to 55.7)*	*(11.77, 30, 8 to 36)*	*(1.02, 4, 2 to 4)*	*(1.72, 4, 2 to 7)*	*(9317.18, 32,756, 14,763.77 to 36,273.98)*	*(3529.22, 8394, 2010.74 to 10,029.17)*	*(4781.22, 16,109, 8318.9 to 20,333.93)*	*(1502.36, 5608, 3323.77 to 8035.16)*	*(1754,67, 4423.66, 1974.88 to 7447.75)*
MIFn	43 years	1.25	1.5	FRI (× 1) NU (× 1) SNU (× 3)	N/A	5.72 cm	12.67 m	2.45	43.67 m	21 days	2	3	£19,037.00	£7460.00	£9049.00	£3122.00	£4532.18
	*(14.11, 39, 29 to 64)*	*(0.5, 1.5, 0 to 3)*	*(0.5, 1.5, 1 to 2)*			*(2.34, 4.95, 3.47 to 9.52)*	*(2.22, 12.52, 4.6 to 15.9)*	*(0.63, 2.8, 1.1 to 2.85)*	*(11.39, 40.22, 12 to 62.43)*	*(3.7, 19, 15 5 to 27)*	*(0.43, 2, 2 to 3)*	*(0.83, 3, 2 to 4)*	*(3283.24, 17,498, 16,535.86 to 29,530.05)*	*(1266.66, 7115, 6247.81 to 12,740.33)*	*(1924.29, 8367, 7213.85 to 15,991.72)*	*(560.13, 3098, 898 to 3930.45)*	*(188,178, 4837.91, 1736.96 to 7030.96)*
*p* value	*0.145*	< *0.001*	*0.012*	*N/A*	*N/A*	*0.435*	*0.092*	*0.647*	*0.866*	*0.775*	< *0.001*	*0.122*	*0.382*	*0.24*	*0.167*	*0.016*	*0.974*
ILFa	40 years	0.2	1.4	Motorcycle (× 3) Pedestrian (× 1) Car occupant (× 1)	12	5.42 cm	14.49 m	2.41	32.45 m	19 days	3	4	£25,364.00	£6906.00	£13,695.00	£4763.00	£4776.16
	*(14.57, 42, 23 to 56)*	*(0.4, 0, 0 to 1)*	*(0.49, 1, 1 to 2)*		*(5.2, 9, 9 to 22)*	*(0.81, 5.15, 4.76 to 7)*	*(3.19, 15.77, 8.3 to 17.27)*	*(0.7, 2.03, 1.58 to 3.52)*	*(21.17, 23.67, 14.97 to 74)*	*(3.31, 20, 13 to 20)*	*(1.17, 3, 1 to 4)*	*(0.63, 4, 3 to 5)*	*(3695.96, 24,621, 20,468.6 to 29,618.24)*	*(3270.29, 5806, 4199.43 to 13,289.08)*	*(1640.32, 14,335, 11,606.84 to 15,598.08)*	*(2220.27, 4377, 1947 to 8771.85)*	*(959.43, 4300.13, 3517.22 to 6044.54)*
ILFn	35 years	0	1	FRI (× 3) NU (× 2)	N/A	6.5 cm	16.5 m	2.3	33.92 m	23 days	4	4	£26,889.00	£6481.00	£14,480.00	£5717.00	£4448.14
	*(8.47, 33, 26 to 47)*	*0*	*(0, 1, 1 to 1)*			*(2.12, 8.1, 3.3 to 8.3)*	*(4.14, 15.33, 12.1 to 22.2)*	*(0.8. 2.2, 1.5 to 3.76)*	*(11.39, 27.6, 25.47 to 55.7)*	*(11.77, 30, 8 to 36)*	*(1.02, 4, 2 to 4)*	*(1.72, 4, 2 to 7)*	*(9317.18, 32,756, 14,763.77 to 36,273.98)*	*(3529.22, 8394, 2010.74 to 10,029.17)*	*(4781.22, 16,109, 8318.9 to 20,333.93)*	*(1502.36, 5608, 3323.77 to 8035.16)*	*(1754.67, 4423.66, 1974.88 to 7447.75)*
*p* value	*0.364*	*0.148*	*0.03*	*N/A*	*N/A*	*0.505*	*0.452*	*0.747*	*0.575*	*0.874*	*0.057*	*1*	*0.959*	*0.62*	*1*	*0.893*	*0.564*
MIFa	38 years	0.8	1.6	Fall > 2 m (× 3) Pedestrian (× 1) Car occupant (× 1)	13	4.74 cm	7.91 m	1.99	14.69 m	17 days	2	3	£17,407.00	£7336.00	£8703.00	£1368.00	£3972.36
	*(13.57, 34, 20 to 57)*	*(1.17, 0, 0 to 3)*	*(0.8, 1, 1 to 3)*		*(7.04, 9, 9 to 27)*	*(1.6, 4.26, 2.2 to 7.6)*	*(1.17, 7.1, 6.97 to 9.97)*	*(0.48, 1.94, 1.35 to 2.73)*	*(3.58, 13, 13 to 21.83)*	*(6.01, 16, 10 to 28)*	*(0, 2, 2 to 2)*	*(0.49, 3, 2 to 3)*	*(4677.6, 15,767, 13,459.45 to 26,461.9)*	*(6029.25, 4480, 3499.45 to 19,349.86)*	*(1660.28, 9813, 6222.84 to 10,177.25)*	*(532.89, 1109, 821.2 to 2034.42)*	*(1114.07, 3972.46, 1959.75 to 5176.71)*
MIFn	43 years	1.25	1.5	FRI (× 1) NU (× 1) SNU (× 3)	N/A	5.72 cm	12.67 m	2.45	43.67 m	21 days	2	3	£19,037.00	£7460.00	£9049.00	£3122.00	£4532.18
	*(14.11, 39, 29 to 64)*	*(0.5, 1.5, 0 to 3)*	*(0.5, 1.5, 1 to 2)*			*(2.34, 4.95, 3.47 to 9.52)*	*(2.22, 12.52, 4.6 to 15.9)*	*(0.63, 2.8, 1.1 to 2.85)*	*(11.39, 40.22, 12 to 62.43)*	*(3.7, 19, 15 5 to 27)*	*(0.43, 2, 2 to 3)*	*(0.83, 3, 2 to 4)*	*(3283.24, 17,498, 16,535.86 to 29,530.05)*	*(1266.66, 7115, 6247.81 to 12,740.33)*	*(1924.29, 8367, 7213.85 to 15,991.72)*	*(560.13, 3098, 898 to 3930.45)*	*(1881.78, 4837.91, 1736.96 to 7030.96)*
*p* value	*0.43*	*0.285*	*0.742*	*N/A*	*N/A*	*0.603*	*0.366*	*0.08*	*0.044*	*0.121*	*1*	*1*	*0.215*	*0.3*	*0.383*	*0.065*	*0.803*

*ASA* American Society of Anaesthesiologists physical status score, *cm* centimetre, *ISS* injury severity score, *FRI* fracture-related infection, ILFa Ilizarov fixation of an acute defect, *ILFn* Ilizarov fixation of a nonunion defect, *LOS* length of stay, *m* months, *MIFa* Masquelet and internal fixation of an acute defect, *MIFn* Masquelet and internal fixation of a nonunion/FRI defect, *N/A* not applicable, *NU* nonunion, *OR* operation room, *SD* standard deviation, *SNU* septic nonunion

**Fig. 1:**
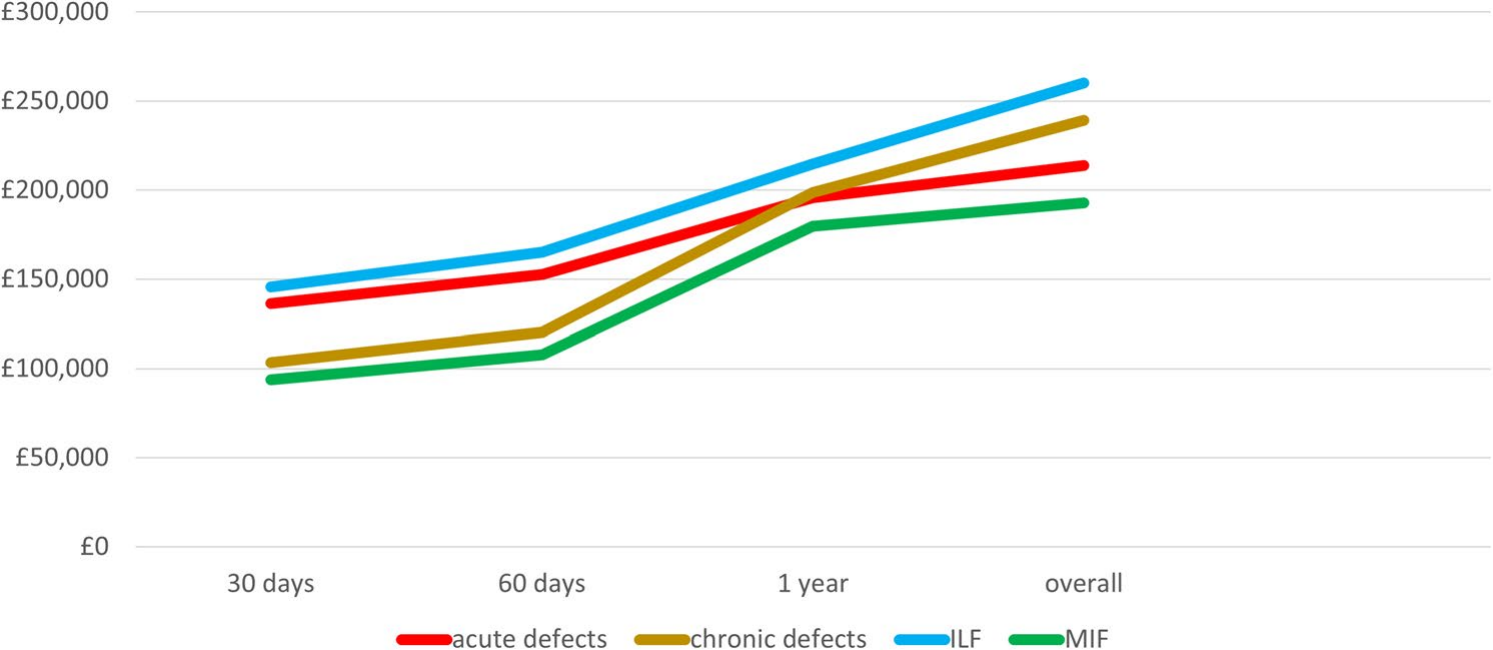
Evolution of the calculated costs between different time intervals (at 30 days, 2 months, and 12 months) from the primary debridement and defect relevant procedure between the different patient groups (acute, nonunion) and the two different methods of treatment (Ilizarov, Masquelet)

The evolution of the imposed costs per method and causative factor is presented in Fig. 1 and Table 5.

**Table 5:** Evolution of the calculated costs between different time intervals from the primary debridement and defect relevant procedure between the different patient groups and the two different methods of treatment

Costs				
Time period		Acute defects (× 10 patients)	Nonunion defects (× 10 patients)	Comparison of mean costs of acute vs. chronic *p* value
30 days post admission	Mean, (SD)	£13,624, (£4542)	£10,070, (£4451)	0.123
	Median, (range)	£13,104, (£5291–£20,940)	£8573, (£4368–£18,735)	
	Sum, (% to overall)	£136,242, (63.7%)	£103,263, (43.2%)	
60 days post admission	Mean, (SD)	£15,254, (£5028)	£11, 541, (£4633)	0.195
	Median, (range)	£16,255, (£5704–£21,837)	£10,782, (£4834–£19,743)	
	Sum, (% to overall)	£152,541, (71.3%)	£120,235, (50.3%)	
1 year post admission	Mean, (SD)	£19,586, (£4372)	£18,933, (£5.343)	0.934
	Median, (range)	£19,598, (£13,215–£26,216)	£16.936, (£12,419–£28,201)	
	Sum, (% to overall)	£195,862, (91.6%)	£198.598, (83.1%)	
Till completion of follow-up	Mean, (SD)	£21,385, (£5796)	£23,399, (£8261)	0.537
	Median, (range)	£21,504, (£13,459–£29,618)	£18,208, (£14,764–£36,274)	
	Sum, (% to overall)	£213,852, (100%)	£239,121, (100%)	

Time period		Distraction osteogenesis protocol (ILF) (× 10 patients)	Induced membrane protocol (MIF) (× 10 patients)	Comparison of mean costs of ILF vs. MIF *p* value
30 days post admission	Mean, (SD)	£14,561, (£4133)	£9390, (£3747)	0.01
	Median, (range)	£15,192, (£8573–£20,940)	£9015, (£4368–£17,200)	
	Sum, (% to overall)	£145,607, (55.9%)	£93,898, (48.7%)	
60 days post admission	Mean, (SD)	£16,509, (£4220)	£10,769, (£4200)	0.01
	median, (range)	£18,063, (£9261–£21,387)	£10,132, (£4833–£17,320)	
	Sum, (% to overall)	£165,087, (63.4%)	£107,689, (55.9%)	
1 year post admission	Mean, (SD)	£21,472, (£4482)	£17,974, (£5135)	0.101
	Median, (range)	£22,715, (£12,897–£26,696)	£16,257, (£12,419–£28,201)	
	Sum, (% to overall)	£214,715, (82.5%)	£179,743, (93.3%)	
Till completion of follow-up	Mean, (SD)	£26,126, (£7126)	£18,131, (£4196)	0.024
	Median, (range)	£27,096, (£14,764–£36,274)	£16,788, (£13,459–£29,530)	
	Sum, (% to overall)	£260,263, (100%)	£192,711, (100%)	

*ILF* Ilizarov frame–distraction osteogenesis, *MIF* two-staged Masquelet protocol with internal fixation and grafting, *SD* standard deviation

## Discussion

The complexity of managing large bone defects is well-described in the literature, as well as the various methods of treatment [2, 3], and their results. [30–32] However, evidence on the health economic aspect of their effective management is extremely scarce [33]. Theoretically, a complete health economic analysis includes direct, indirect, and intangible costs [4], whereas a cost effectiveness study should address both the societal and the health-care payer perspectives evaluating all relevant costs and benefits to the patient over their lifetime [34, 35].

Recently, Norris et al. [33] published a database analysis utilizing two different US-based sources including 904 patients with either the diagnosis of fracture/nonunion/osteomyelitis, treated with bone graft, cement spacer, or a frame fixator. Payer costs were analysed from the index admission to 12 months postoperatively. They concluded that patients with large defects require extended therapies, multiple hospital visits and admissions, representing a significant financial challenge.

Limb reconstruction procedures (ILF and MIF) are considered discrete episodes of care, associated with high up-front costs [33]. With this pilot cost analysis, we aimed to explore the differences of direct medical costs of the two main methods of managing acute or nonunion tibial defects in the best-case scenario of a successful union.

Within the limitations of our study, we recognise that we analysed a small number of patients (type II error). The size of our sample was not based on statistical power calculations, as the scope of this pilot study was to show the feasibility of collecting the data for conducting robust and detailed cost analysis and inform future evaluations of costs and effectiveness. The small number of patients in each subgroup prevented us from adjusting for clinical differences in terms of gender, Charlson’s score, etc. Since we studied a representative sample of patients with successful defect union (best-case scenario), our means and standard deviations may be artificially small, whilst we have compared values following their log transformation to address skewness. According to the power calculation based on the data herein, 35 patients from each group at a 1:1 ratio will be required to detect a 25% difference, with an alpha value of 0.05 and probability (power) of 0.9.

This study does refer to patients with complete data and a successful discharge following healing of their tibial defect. All possible direct medical costs during the initial treatment period, outpatient care, readmissions, and reoperations were measured. Exceptions were costs of outpatient rehabilitation, medications prescribed from primary care or purchased privately, and those of outpatient-parenteral-antibiotic-therapy services (OPAT), as well as productivity losses relevant to time off work. Noteworthy, the absence of health-related quality-of-life measures in this series, as well as the lack of adequate data in the literature, does not allow the comparison in QALY terms, but only into numerical figures of these direct medical costs.

The described clinical results in our series were found to be in accordance with other similar series for both the ILF [6, 36–39] and MIF [26, 30, 40, 41] methods. The demographics and bone defect size, the mean healing index of 2.1, and the incidence of complications per Paley classification [42] of the 5 ILFn patients in this study are consistent with those in the series of Krappinger et al. [38] Similarly, the baseline characteristics and overall outcome (mean healing index of 2.2 months/cm) reported by Mekhail et al.[39] were comparable to our subgroup of ILFa patients.

Main contributors on the cost differences noted (Tables 3, 4) were those related to the OR, and the more intense follow-up ILF patients require till defect union and consolidation of the regenerate bone. This is consistent with existing meta-analysis studies [30–32]. Selection bias between the groups is possible, as patients were not randomised preoperatively to receive either of the two methods.

The comparison between acute and nonunion/infected defects revealed lower number of admissions (*p* = 0.036), shorter follow-up (*p* = 0.04), and time-to-union for the acute defects (*p* = 0.048). No statistically significant differences were observed for the cost of infected cases (*p* = 0.537). This is perhaps attributable to the relatively low costs of the antibiotic therapies and the fact that it was not possible to capture costs incurred by primary care providers (including those of the OPAT service).

There is clear need for a pivotal health economic evaluation in this area, utilising the findings and some of the methodological aspects of the current study. The absolute need of using a health-related quality-of-life score as utility measures in future clinical series is also apparent to facilitate the translation of patient reported outcomes into effectiveness measures that are adequate to inform the optimal allocation of the scarce healthcare resources [34, 35].

Currently, in the NHS, limb reconstruction belongs to the specialist high-cost-tariff-excluded devices (HCTED) [43], attracting certain uplifts to their reimbursement. The generation of robust health economic evidence is expected to facilitate the update of such reimbursement arrangements, and their adoption into those managed with different techniques, as the Masquelet method.

This study does not report on the exact revenue of our unit, as this is influenced from the reimbursement arrangements of our hospital, and the reduced prices following the local implant tender. To provide more generalizable evidence, which could be relevant to different clinical groups, we based all our study on generic price lists and cost values, which do not take into account local negotiated prices.

The clinical need to have both methods available, together with others, is apparent from their widespread use globally. Each technique provides different features and advantages which make them preferable to certain scenarios. Bone transport (ILF) has many proven advantages in complex defects with associated deformities, allowing simultaneous tackling of all associated problems (bone defect reconstruction, realignment, infection control, mechanical stability, and immediate mobilisation) [6, 37, 44]. The more recently introduced Masquelet technique offers similar advantages and successful defect management independent of defect size. In addition, it requires less intense follow-up and probably is better suited for less compliant patients. [5, 40, 41]

## Conclusion

This series of patients represents the routine experience of a large limb reconstruction trauma centre, utilising a variety of complementary methods to address the challenge of bone defect reconstruction. The results and analysis presented lead to some preliminary evidence on factors affecting the financial burden that such centres face. This highlights the need for further larger and more complete studies to aid decision makers and clinicians to improve contemporary reimbursement policies, ensuring that complex bone defect reconstruction is appropriately supported.

## Data availability statement

Data supporting the reported results can be found to the Trauma-Related Services CSU clinical audit database of Leeds Teaching Hospitals NHS Trust.
